# Defective Granuloma Formation in Elderly Infected Patients

**DOI:** 10.3389/fcimb.2020.00189

**Published:** 2020-04-29

**Authors:** Aurélie Daumas, Benjamin Coiffard, Céline Chartier, Amira Ben amara, Julie Alingrin, Patrick Villani, Jean-Louis Mege

**Affiliations:** ^1^Aix-Marseille Univ, IRD, Assistance Publique-Hôpitaux de Marseille (APHM), MEPHI, IHU-Méditerranée Infection, Marseille, France; ^2^Service de Médecine Interne, Gériatrie et Thérapeutique, Hôpital de la Timone, APHM, Marseille, France; ^3^Service d'Anesthésie et de Réanimation, Hôpital Nord, APHM, Marseille, France

**Keywords:** sepsis, elderly patient, granuloma, tumor necrosis factor, multinucleated giant cell

## Abstract

Granulomas are compact structures formed in tissues by the immune system in response to aggressions. The *in vitro* formation of granulomas using circulating mononuclear cells is an innovative method to easily assess the immune response of patients. Monitoring the efficiency of mononuclear cells from patients to form granulomas *in vitro* would help improve their therapeutic management. Circulating mononuclear cells from 23 elderly patients with sepsis and 24 elderly controls patients were incubated with Sepharose beads coated with either BCG or *Coxiella burnetii* extracts. The formation of granulomas was measured over 9 days. Most healthy elderly patients (92%) were able to form granulomas in response to BCG and *Coxiella burnetii* extracts compared to only 48% of infected elderly patients. Undernutrition was significantly associated with impaired granuloma formation in healthy and infected patients. Granulomas typically comprise epithelioid cells and multinucleated giant cells, however, these cells were not detected in samples obtained from patients unable to form granulomas. We also found that the impairment of granuloma formation was associated with reduced production of tumor necrosis factor without overproduction of interleukin-10. Finally, all genes specifically modulated in granulomatous cells were down-modulated in patients with defective granuloma formation. TNFSF10 was the only M1 gene markedly upregulated in patients who did not form granulomas. Our study suggest that defective granuloma formation may be a measurement of altered activation of immune cells which can predispose to nosocomial infections in elderly patients.

## Introduction

Granulomas are organized collections of immune cells that reflect tissue immune response to aggressions (Saunders and Britton, 2007; Herndon and Rogers, 2013; Pagán and Ramakrishnan, 2018). They are dynamic structures based on the recruitment of monocytes, macrophages, and T lymphocytes, and the subsequent differentiation of macrophages into epithelioid cells and multinuclear giant cells (MGCs) (Saunders and Britton, 2007; Herndon and Rogers, 2013). One of the main features of the immune response to *Mycobacterium Tuberculosis* and the outcome of Q fever caused by *Coxiella burnetii* is the formation of granulomas (Saunders and Britton, 2007; Herndon and Rogers, 2013). Granulomas have a significant protective function (Ramakrishnan, 2012; Herndon and Rogers, 2013; Pagán and Ramakrishnan, 2018). Their main function is to isolate bacteria or other pathogens from the body and facilitate their destruction by granulomatous cells (Delaby et al., 2012; Ramakrishnan, 2012; Pagán and Ramakrishnan, 2018). In tuberculosis, granuloma formation is primarily a host-defense mechanism for containing the bacteria. However, some bacilli can survive inside these structures which providing them a niche, up to a potential reactivation (Saunders and Britton, 2007; Ramakrishnan, 2012; Silva Miranda et al., 2012). Granulomas are present in patients with acute Q fever but the defective immune response observed in chronic Q fever is associated with the absence of granulomas (Delaby et al., 2012; Eldin et al., 2017). Cytokines and chemokines play a critical role in granuloma formation through their control of the recruitment and activation of immune cells (Turner et al., 2014). Indeed, type 1 cytokines such as interferon (IFN)-γ, tumor necrosis factor (TNF), and interleukin (IL)-12 are involved in the acquisition of the microbial competence of macrophages and contribute substantially to the ability of the host to eradicate pathogens. Conversely, IL-10, an anti-inflammatory cytokine, negatively regulates the protective immune response (Gallegos et al., 2011). M1 macrophages are induced by IFN-γ with or without pathogen-associated patterns, they are characterized by increased expression of Toll-like receptors, MHC class II, production of inflammatory cytokines and oxygen derivatives; they promote Th1 responses, microbicidal and tumoricidal activities. In contrast, M2 macrophages are induced by IL-4 or IL-13, express a large panel of C-type lectins, MHC class II and CD163; they express arginase and anti-inflammatory cytokines, promote Th2 responses, parasite clearance and inflammation termination (Murray et al., 2014). In intracellular bacterial infections, CD4^+^ T cells differentiate into T helper type 1 (Th1) effector cells that secrete IFN-γ and TNF; these mediate protection by stimulating the anti-microbial activity of macrophages (Saunders and Britton, 2007). In a mouse model of mycobacterial infection, the absence of TNF and IFN-γ leads to impaired granuloma formation and increases bacterial infection (Beham et al., 2011; Gallegos et al., 2011). Furthermore, the use of anti-TNF antibodies in patients highlights the role of TNF in granuloma formation. Clinical observations have revealed that anti-TNF-α treatment is associated with a risk of tuberculosis reactivation. However, we recently reported that anti-TNF antibodies do not affect the formation of granulomas but that of MGCs (Mezouar et al., 2019). In humans, impaired IL12/IFN-γ predisposes patients to mycobacterial infections and interferes with granuloma formation depending on the severity of IFN-γ impairment (Naranbhai, 2016).

The study of tissue granulomas in patients requires biopsies, which cannot be performed routinely. Recently, an *in vitro* alternative was proposed. The method is based on the culture of peripheral blood mononuclear cells (PBMCs) with Sepharose beads coated with extracts from BCG, an attenuated strain of *Mycobacterium bovis* (Puissegur et al., 2004), and from *Coxiella burnetii* (CB) (Delaby et al., 2010). The *in vitro* formation of these cell culture systems provides an easy mean for studying the coordination of innate and adaptive immunity that is not possible in patients and may be difficult in animal models. This method is convenient to study the initial phases of granuloma formation and the transcriptional signature. More than 50% of genes are commonly modulated in response to CB and BCG. They include M1-related genes such as HESX1, TNFSF10, IDO1, and TNF, and genes related to chemotaxis (CCL2, CCL5). CB strongly upmodulates the expression of genes involved in microbicidal response, especially ISGs including IFIT1. Furthermore, the expression of genes such as FASLG and GNLY involved in cell death is increased in response to BCG (Faugaret et al., 2014). This approach enables the formation of granulomas to be investigated in clinical practice. Indeed, the *in vitro* formation of granulomas is defective in the majority of patients with chronic Q fever due to a lack of migration of monocytes toward CB-coated beads (Delaby et al., 2012). In brain injury patients, the defective *in vitro* formation of granulomas involving monocytes, natural killer cells, and γδ T cells, is associated with increased nosocomial pneumonia (Deknuydt et al., 2013). In patients with severe sepsis, the defective *in vitro* formation of granulomas is associated to monocytopenia and a reduced production of TNF (Alingrin et al., 2016).

Elderly individuals are at risk of contracting infectious diseases due to their declining immune system known as “immunosenescence.” If the latter affects both innate and adaptive immunity, other factors likely contribute to the increased risk; these include undernutrition, comorbidities, altered mucosal barriers, decreased cough reflex, and changes in the urinary tract (Gavazzi and Krause, 2002; Hepper et al., 2013). The most common comorbidities associated to an increased risk of infection are congestive heart failure, chronic kidney failure, diabetes mellitus, cirrhosis, chronic obstructive lung disease, and malignancies (Esper et al., 2006). In infectious diseases, patient outcome is dependent by a complex interplay between pro- and anti-inflammatory host responses (Faix, 2013). In most patients, the pro-inflammatory response is self-limited. However, in patients in sepsis, the response is exaggerated and leads to a compensatory downregulation of the immune system during which the patient is susceptible to organ dysfunction and nosocomial infection. The mechanisms of resistance or susceptibility of individuals are not well-understood. A major risk factor seems to be a pre-existing immune dysfunction. For instance, elderly patients and immunosuppressed patients have a higher incidence of sepsis and a higher mortality rate (Faix, 2013). Martin et al. (2006) showed that the incidence of sepsis is higher in elderly adults and age is an independent predictor of mortality.

We hypothesized that the efficiency of mononuclear cells from infected patients to form granulomas *in vitro* was lower compared to elderly controls which can predispose to nosocomial infections. To test this hypothesis, we assessed the ability of PBMCs to generate granulomas *in vitro* in response to BCG and CB, in order to limit a possible bias linked to our prior immunization (a majority vs. a minority of immunized patients).

## Materials and Methods

### Study Population

Patients recruitment was provided from an ancillary study to NCT02734017. Written informed consent was obtained from the patients or their relatives in accordance with the Declaration of Helsinki. Patients from the geriatric unit at Timone Hospital in Marseille, France were enrolled from January 1st 2017 to April 30th 2017 according to the following criteria: aged over 65 years with at least three chronic illnesses or over 75 years and with presence or absence of sepsis without organ failure lasting for <48 h requiring empirical antimicrobial treatment. We included 23 infected patients with different pathogens and sources of infection and 24 patients without infection as controls. The latter were devoid of immunodeficiency, cancer in the previous 5 years, and immunosuppressive and immunomodulatory drugs. At the time of blood collection, we also collected demographic variables, the body mass index (BMI) to identify undernourished patients (BMI <21, threshold set for the elderly by the French National Health Authority), comorbidities, biological data, and the source of infection for the infected patients. The main clinical characteristics of the 47 patients enrolled are summarized in Table 1.

**Table 1:** Characteristics of the study population.

	**Patients with infection (*n* = 23)**	**Patients without infection (*n* = 24)**	***p***
**Age (years)**			
mean (min-max)	81.7 (62–100)	84.6 (74–100)	0.78
**Male**, ***n*** **(%)**	9 (39.1)	11 (45.8)	0.25
**Medical history**, ***n*** **(%)**			
Diabetes Mellitus	10 (43.5)	4 (16.6)	0.06
Undernutrition (BMI <21)	4 (17.4)	1 (4.2)	0.16
Chronic lung disease	1 (4.3)	2 (8.3)	0.56
Cardiac insufficiency	6 (26.1)	7 (29.2)	0.75
Dementia	5 (21.7)	9 (37.5)	0.21
**Number of circulating cells**			
**on admission**			
**Leukocytes (Giga/L)**			
mean [min-max]	10.3 [4.4–20]	6.7 [4.4–11]	<0.001^*^
**Lymphocytes (Giga/L)**			
mean [min-max]	1.3 [0.44–3.6]	1.7 [0.62–3.6]	0.09
**Neutrophils (Giga/L)**			
mean [min-max]	8.1 [3–17]	4.4 [2.1–9.6]	<0.001^*^
**Monocytes (Giga/L)**			
mean [min-max]	0.7 [0.21–1.6]	0.7 [0.22–3.8]	0.75
**CRP (mg/L)**			
mean [min-max]	163.3 [10–449.8]	10.1 [0–48]	<0.001^*^
**Albumin (g/L)**			
mean [min-max]	28.7 [18–41.8]	36.1 [29–44.3]	<0.001^*^

*p values for comparisons between patients with infection and healthy controls*.

* *p < 0.05*.

### Mononuclear Cell Isolation

EDTA-anticoagulated blood samples (5 mL) were collected (on day of initiation of empirical antibiotic treatment for infected patients) and immediately sent to the laboratory. Peripheral blood mononuclear cells (PBMCs) from infected patients and controls were isolated from whole blood using a Ficoll gradient (MSL, Eurobio) and suspended in RPMI 1640 containing 25 mM HEPES and 2 mM L-glutamine (Invitrogen) (Alingrin et al., 2016). After centrifugation at 500 × g, PBMCs were washed in sterile phosphate-buffered saline (PBS, Life Technologies) and suspended (about 5 × 10^6^ cells/ml) in RPMI 1640 supplemented with 20% fetal calf serum (FCS, Invitrogen) and 10% dimethylsulfoxide, and preserved at −80°C.

### Coupling of Beads With Bacterial Extracts

CB organisms (Nine Mile strain, RSA 495) and BCG (CIP 105050) were cultured as described previously (Delaby et al., 2010). The bacteria (10^9^ per assay) were sonicated in a coupling buffer (0.1 M NaHCO3 pH 8.3 containing 0.5 M NaCl) and their protein content was determined using Bradford's method (BioRad protein assay) (Delaby et al., 2010). Cyanogen-bromide (CN-Br)-activated Sepharose 4B beads 40 to 100 μm in diameter (GE Healthcare, France) were suspended in 1 mM HCl for 15 min and then washed in a coupling buffer according to the manufacturer's instructions.

Bacterial extracts (0.5 mg of protein corresponding to about 2.5 × 10^8^ bacteria) were added to 10 mg (4 × 10^4^ beads) of beads and mixed overnight at 4°C in a coupling buffer. After centrifugation at 120 × g for 10 min, the coupling efficiency of the bacterial extracts was determined by measuring the protein content of the supernatants. The beads were then washed, and the remaining CN-Br active groups were blocked by incubating the beads with 0.1 M Tris–HCl buffer pH 8.0 for 2 h. After centrifugation at 120 × *g* for 10 min, the beads were washed again three times in acetate and Tris-HCl buffers. The beads were finally stored at 4 °C in PBS for 1 month.

### Granuloma Formation

*In vitro* granuloma formation was assessed after incubation of 2.5 × 10^5^ PBMCs with 50 Sepharose beads coated with either CB or BCG extracts in 96-well plates containing RPMI 1640 medium supplemented with 25 mM HEPES, 2 mM of L-glutamine, 10% FCS, and antibiotics at 37°C. The formation of granulomas was evaluated after 3, 6, and 9 days of culture using inverse microscopy (DIM3000 B, Leica). Only beads completely covered with cells were considered granulomas. The percentage of beads with granulomas was determined by optical microscope examination of 3 wells per experiment.

### Cell Characterization

Granulomas were collected with manual pipette. They were then incubated with 0.05% Trypsine-0.53Mm EDTA for 15 min at 37°C to dissociate cells. Cells were collected, plated onto glass slides with a cytospin (5 min at 1,500 rpm) and then stained with May-Grünwald Giemsa (Sigma, France) to identify epithelioid cells and MGCs in dissociated granuloma cells (Puissegur et al., 2004; Delaby et al., 2010). MGCs were identified by the presence of more than three nuclei. The percentage of epithelioid cells and MGCs in the stained preparations was quantified by optical microscope examination after 3, 6, and 9 days of culture.

Dissociated granuloma cells (5 × 10^5^) were also analyzed using flow cytometry (Puissegur et al., 2004; Delaby et al., 2010). PBMCs were labeled with a mixture of the following fluorescent antibodies: CD3-PC5 (Beckman Coulter, France), CD4-APC (Dako, France), CD8-PE (Beckman Coulter, France), CD68-FTIC (Dako, France), and CD45-APC H7 (Becton Dickinson Biosciences, France) with isotype-matched fluorophore-conjugated immunoglobulin G (IgG) for the controls. Cell populations were identified using a CANTO II flow cytometer (Becton Dickinson Biosciences) and DIVA BD software (San Jose, CA) was used to analyze the data.

In patients unable to form granulomas, the cells were recovered without having to dissociate them from the beads and analyzed in the same way as the granuloma cells with May-Grünwald Giemsa staining and immunophenotyping.

### Cytokine Measurement

PBMCs (2.5 × 10^5^ cells/well) were incubated with coated beads (50 beads/well) and the supernatants were collected after 1 and 3 days, whether or not granulomas have formed. TNF and IL-10 production were measured in the supernatants from cell cultures forming or not granulomas, using enzyme immunoassays (R&D Systems, Quantinine® ELISA kit) according to the manufacturer's instructions. The results were expressed as pg/ml. The intra- and inter-specific coefficients of variation ranged from 5 to 10%.

### Transcriptomic Analysis (RNA Extraction and qRT-PCR)

Granuloma cells were dissociated by incubation in PBS buffer containing 2 mM EDTA (Invitrogen) if necessary. Total RNA from granuloma cells or mononuclear cell was extracted and treated with DNase using the RNeasy® Mini Kit (Qiagen). Reverse transcription of 150 ng of total RNA was performed as described previously (Ben Amara et al., 2010). We assessed the expression of nine M1 (TNFSF10, IL15RA, IDO1, IL2RA, TNF, IL15, EDN1, HESX1, CXCL29), seven M2 (ALOX15, FN1, CCL23, CCL13, CLEC4, CSTE, HRH1), and four granulomatous genes (EPBH2, FASLG, GNLY, IFIT1) in response to CB and BCG, as previously studied (Faugaret et al., 2014). Gene expression was analyzed in cells from granulomas after dissociation in eight individuals (5 were infected) who formed granulomas and in mononuclear cells in three patients who did not form granulomas (2 were infected). PBMCS were cultured without coated beads (unstimulated cells) and were used as control for fold change calculation. All selected primers were designed using Primer3 (version 0.4.0; http://bioinfo.ut.ee/primer3/). Quantitative PCR was performed using LightCycler FastStart DNA Master^PLUS^ SYBR Green I (Roche). The results were normalized with the housekeeping gene β-actin. The fold change (FC) of the target genes relative to β-actin was computed using the formula FC = 2^−ΔΔCt^, where ΔΔCt = (Ct_Target_ - Ct_Actin_)_stimulated_ - (Ct_Target_ - Ct_Actin_)_unstimulated_ (Ben Amara et al., 2010).

### Statistical Analysis

Quantitative data are presented as the mean with range or standard deviation. Qualitative results are presented as absolute counts and percentages. Results between groups were compared using Student's *t*-test or Mann-Whitney non-parametric test when the conditions for applying the *t*-test were not met. Differences were considered significant when *p* < 0.05. Data analysis was performed, and plots were generated using GraphPad Prism 5 (GraphPad Software Inc).

## Results

### Patient Characteristics

The characteristics of the study population are shown in Table 1. Twenty-three infected patients (14 women and 9 men) with an average age of 81.7 years and 24 uninfected controls (13 women and 11 men) with an average age of 84.6 years were included. The leukocyte and neutrophil counts were significantly (*p* < *0.001*) higher in infected patients than in controls, whereas the monocyte and lymphocyte counts were not significantly different. C-reactive protein and albumin levels were significantly (*p* < *0.001*) higher and lower, respectively, in infected patients. The most frequent infections were urinary tract infections (7 patients) and pneumonia (7 patients). The microbial etiology was identified in <50% of infected patients and consisted mainly of gram-negative bacilli (*n* = 8). No patient had a medical history of tuberculosis or Q fever.

### Defective Formation of Granulomas in Infected Patients

**Figure 1:**
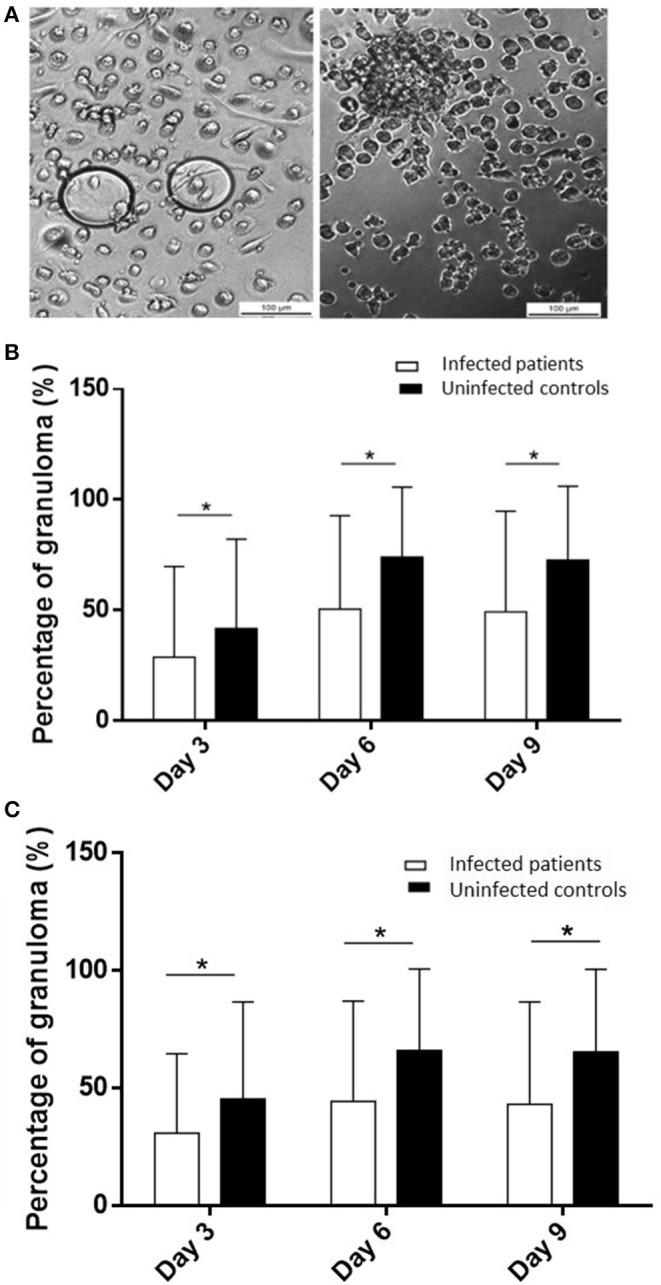
Granuloma formation in infected patients and uninfected controls. PBMCs (2.5 × 10^5^) from healthy and infected patients were incubated with 50 Sepharose beads coated with bacterial extracts for 9 days. The entire content of three wells per experiment was examined under an optical microscope to determine the number of granulomas. Only beads completely covered by cells were considered granulomas. **(A)** Representative micrographs of beads coated with BCG with PBMCs from an infected patient with no granuloma (left) and from a control with a representative granuloma (right). **(B,C)** The number of generated granulomas in response to BCG extracts **(B)** and CB extracts **(C)** was enumerated and the results are expressed in percentage of bead-associated granulomas and presented as the mean ± SD. *p < 0.05.

**Figure 2:**
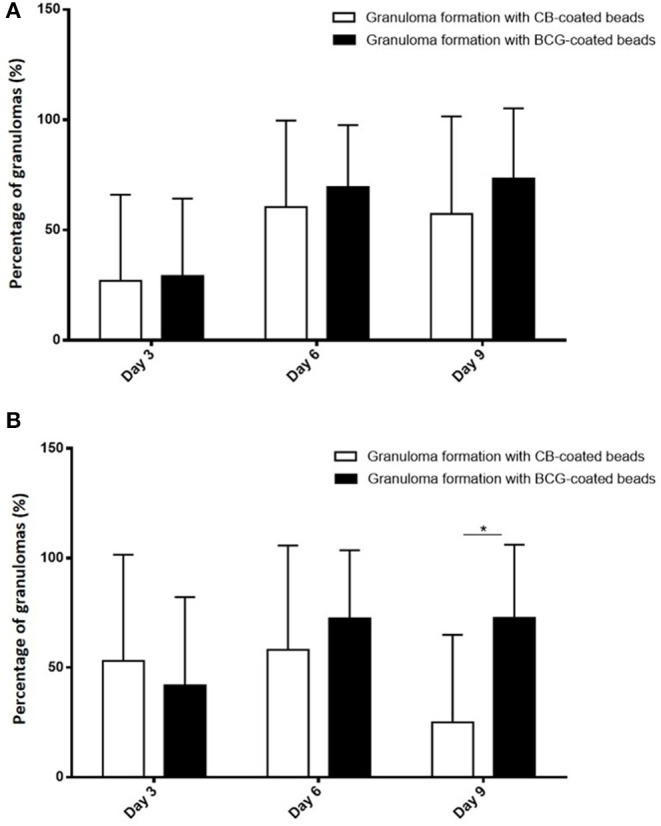
Comparison of granuloma formation in response to BCG and CB extract. PBMCs (2.5 × 10^5^) from healthy and infected patients were incubated with 50 Sepharose beads coated with bacterial extracts for 9 days. The entire content of three wells per experiment was examined under an optical microscope to determine the number of granulomas. Only beads completely covered by cells were considered granulomas. The results are expressed in percentage of bead-associated granulomas and presented as the mean ± SD. *p < 0.05. **(A)** There was no significant difference in granuloma formation in infected patients, regardless of the beads used. **(B)** In **controls**, the percentage of granulomas on day 9 was significantly lower with the CB-coated beads than the BCG-coated beads.

To test if the occurrence of infection in elderly subjects was associated with impaired granuloma formation, PBMCs from controls and infected patients were incubated with beads coated with BCG and granuloma formation was measured for 9 days (Figure 1A). The great majority of uninfected controls (22 out of 24, 92%) were able to form granulomas, whereas only 48% of infected patients were. The impaired formation of granulomas in infected patients was not due to a delayed formation because the formation of granulomas was significantly lower in infected patients than in controls at days 3, 6, and 9 (*p* < 0.05) (Figure 1B). After cultivating PBMCs with BCG-coated beads for 3 days, the formation of granulomas was significantly lower in infected patients (22 ± 36%), whereas it reached 42 ± 40% in controls. It was not a delayed formation because it remained lower in infected patients than in controls after 6 days (50 ± 42 vs. 74 ± 31%) and 9 days (49 ± 45 vs. 72 ± 33%). Interestingly, when CB-coated beads were used instead of BCG-beads, the patients who did not form granulomas in response to BCG extracts were also unable to form granulomas in response to CB extracts. After 3 days of culture of PBMCs with CB-coated beads, the formation of granulomas was also significantly low in infected patients (*p* = 0.03). The formation of granulomas remained lower in infected patients than controls after 6 and 9 days (*p* = 0.0049 and 0.002, respectively) (Figure 1C). The comparison of granuloma formation in response to BCG and CB extracts did not show any significant difference, except at day 9 for controls for whom the granuloma percentage was significantly lower in response to CB extracts than in response to BCG (Figure 2). Taken together, these results show that the ability to develop an *in vitro* granulomatous response to BCG or CB extracts was altered in infected elderly patients. Given these results, the following experiments were performed with BCG-coated beads only.

### Granuloma Formation and Patient Characteristics

As infected patients were partitioned into two populations able or unable to form granulomas, we investigated if impaired granuloma formation was associated with some clinical and/or biological features of patients (Tables 2–4). We found that infected female patients were more prone to forming granulomas than their male counterparts. This gender effect was not observed in controls. Among the clinical parameters, undernutrition defined by a BMI <21 was significantly associated with impaired granuloma formation in both controls and infected patients (*p* = 0.02). The inflammatory status as assessed by the CRP level was not significantly different in infected patients who did or did not form granulomas. Leukocyte and neutrophil counts were significantly higher in controls who formed granulomas than in patients who did not form granulomas. No difference was found in infected patients. So, except undernutrition, no significant difference was found between patients with and without granuloma formation. Hence, clinical and biological parameters were not sufficient to account for impaired granuloma formation.

**Table 2:** Granuloma formation and characteristics of infected patients.

	**Infected patients forming granulomas**	**Infected patients not forming granulomas**	***p***
**Number of patients**	11/23 (48%)	12/23 (52%)	0.77
**Patients (female/male)**	9/2	4/7	1
**Age (years)**			
mean (min-max)	82.9 [65–100]	80.7 [65–93]	0.59
**Medical history, n**			
Diabetes Mellitus	3/11	7/12	0.14
Undernutrition (BMI <21)	0/11	4/12	0.02^*^
Chronic lung disease	0/11	0/12	1
Cardiac insufficiency	3/11	2/12	0.56
Dementia	2/11	4/12	0.43
**Leukocytes (Giga/L)**			
mean [min-max]	12 [5.9–20]	9.2 [4.4–12]	0.5
**Neutrophils (Giga/L)**			
mean [min-max]	9.8 [4.5–17]	7.0 [3–10]	0.7
**Monocytes (Giga/L)**			
mean [min-max]	0.9 [0.33–1.6]	0.6 [0.21–1.3]	0.1
**Lymphocytes (Giga/L)**			
mean [min-max]	1.3 [0.44–3.6]	1.4 [0.8–2.3]	0.8
**C-reactive protein (mg/L)**			
mean [min-max]	195.3 [36.2–449.8]	143.1 [10–337]	0.25
**Albumin (g/L)**			
mean [min-max]	29.5 [19–41.8]	27.9 [18–34.6]	0.57

*p-values for comparisons between infected patients that formed granulomas and infected patients that did not form granulomas*.

* *p < 0.05*.

**Table 3:** Sources of infection and pathogens involved in infected patients.

	**Infected patients forming granulomas**	**Infected patients not forming granulomas**
Sources of infection, *n*	Urinary infection (*n* = 2) Pneumonia (*n* = 3) Digestive infection (*n* = 2) Skin infection (*n* = 2) Spondylitis (*n* = 1) Sepsis (*n* = 1)	Urinary infection (*n* = 5) Pneumonia (*n* = 4) Digestive infection (*n* = 1) Sepsis (*n* = 2)
Pathogens involved, *n*	4/11*E. coli* (*n* = 3) *S. aureus* (*n* = 1)	7/12*E. coli* (*n* = 3) *S. aureus* (*n* = 1) *P. mirabilis* (*n* = 1) *K. pneumoniae* (*n* = 1) Metapneumovirus (*n* = 1)

**Table 4:** Granuloma formation and characteristics of uninfected controls.

	**Controls forming granulomas**	**Controls not forming granulomas**	***p***
**Number of patients**	22/24 (92%)	2/24 (8%)	
**Patients (female/male)**	12/10	2/0	0.23
**Age (years)**			
mean (min-max)	83.1 [74–100]	84 [81–87]	0.82
**Medical history, n**			
Diabetes Mellitus	4/22	0/2	0.53
Undernutrition (BMI <21)	1/22	2/2	0.02 ^*^
Chronic lung disease	2/22	0/2	0.67
Cardiac insufficiency	2/22	0/2	0.67
Dementia	7/22	0/2	0.36
**Leukocytes (Giga/L)**			
mean [min-max]	6.8 [4.4–11]	5.55 [5.4–5.7]	0.002^*^
**Neutrophils (Giga/L)**			
mean [min-max]	4.4 [2.9–9.6]	2.9 [2.1–3.2]	0.02^*^
**Monocytes (Giga/L)**			
mean [min-max]	0.71 [0.29–3.8]	0.4 [0.22–0.61]	0.34
**Lymphocytes (Giga/L)**			
mean [min-max]	1.65 [0.62–3.6]	1.75 [1.2–2.3]	0.89
**C-reactive protein (mg/L)**			
mean [min-max]	8.7 [0–33]	25.0 [2–48]	0.61
**Albumin (g/L)**			
mean [min-max]	36.3 [29–44.3]	34.1 [32.3–36]	0.44

*p-values for comparisons between controls forming granulomas and controls not forming granulomas*.

* *p < 0.05*.

### Cell Composition of Granulomas

We measured the proportion of cell populations placed in culture from patients who would form granulomas and those who would not. The proportion of monocytes and CD4^+^ and CD8^+^ T lymphocytes determined using flow cytometry was similar between controls and infected patients regardless of their capacity to form granulomas or not (data not shown).

In the absence of granuloma formation, the proportion of monocytes and T cells did not change during the culture. In the presence of granuloma formation in response to BCG, CD4^+^ T lymphocytes steadily increased during the culture with BCG-coated beads, whereas CD8^+^ T cells moderately decreased during the same time. No difference was observed between infected and controls. Monocytes from each group decreased during the 9 days of culture; this is related to their macrophage maturation (data not shown).

**Figure 3:**
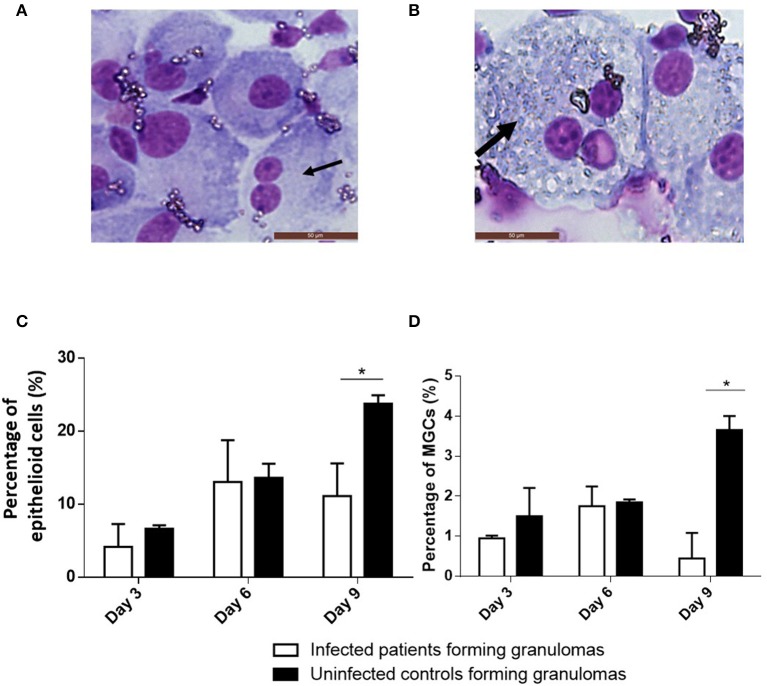
Cell composition of granulomas. BCG-granulomas were recovered and the cells were dissociated from the beads by mechanical agitation. They were then stained using May-Grünwald Giemsa staining and observed under an optical microscope. The percentages of cells were compared between six infected patients and six controls. Each experiment was performed in duplicate. **(A)** The black arrow indicates a typical epithelioid cell. **(B)** The thick black arrow indicates a typical MGC. **(C,D)** The proportion of epithelioid cells and MGCs relative to the granuloma cells was determined after 3, 6, and 9 days. The results are presented as the mean ± SD. *p < 0.05.

As granulomas are known to be rich in epithelioid cells and MGCs (Figures 3A,B), their presence was assessed using May-Grünwald-Giemsa staining after 3, 6, and 9 days. When cultured mononuclear cells were unable to form granulomas, especially in infected patients, neither epithelioid cells nor MGCs were found. When granulomas were formed, epithelioid cells and MGCs were detected from day 3 and reached almost 25 and 4% of granulomatous cells, respectively, on day 9 in controls (Figure 3C). In infected patients, epithelioid cells represented on average 13% of the granuloma cells on day 6 then decreased to reach about 10% after 9 days. Concerning MGCs, they did not exceed 2% after 6 days of culture then decreased (Figure 3D). Thus, granulomas of controls contained significantly more epithelioid cells and MGCs than granulomas of infected patients (*p* = 0.04 and *p* = 0.02, respectively). These results highlighted that the defective formation of granulomas was associated with deficient epithelioid cell and MGC formation.

### Cytokines and Deficient Granuloma Formation in Infected Patients

**Figure 4:**
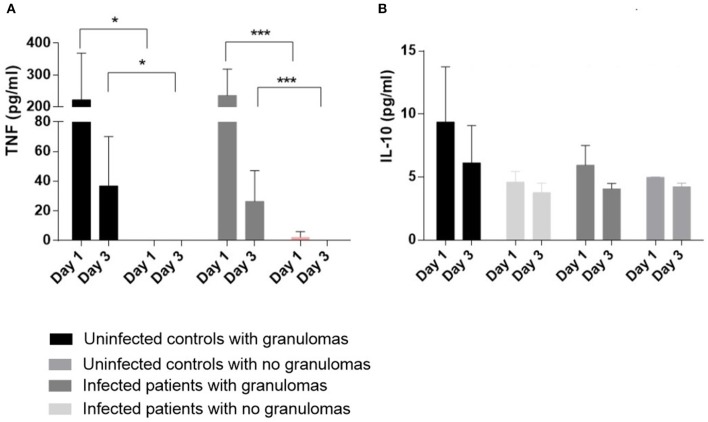
Cytokine production PBMCs (2.5 × 10^5^) from 13 controls (11 who formed granulomas and 2 who did not form granulomas) and 16 infected patients (7 who formed granulomas and 9 unable to form granulomas) were incubated with 50 Sepharose beads coated with BCG extracts for 1 and 3 days. The supernatants were collected after 1 and 3 days. TNF measurements **(A)** and IL-10 measurements **(B)** were performed with the supernatants from cell cultures forming or not granulomas, using enzyme immunoassays. The results are expressed as pg/mL and presented as the mean ± SD. *p < 0.05, ***p < 0.0001.

As the defective formation of granulomas in infected patients may be associated with a decrease in inflammatory cytokines, we measured the release of TNF and IL-10 by cells incubated with BCG-coated beads for 1 and 3 days. First, we noticed that the amounts of TNF in the supernatants were lower on day 3 compared with the results on day 1. The amounts were significantly lower in controls and infected patients who did not form granulomas than in controls and infected patients with granuloma formation on day 1 as well as on day 3 (*p* < 0.05 for controls and *p* < 0.001 for infected patients) (Figure 4). The decrease in production of TNF may be related to an increase of an immunosuppressive cytokine such as IL-10. Therefore, the amounts of IL-10 in the supernatants were measured. They were low and similar in infected and controls with or without granuloma formation (Figure 4). These results suggested that the defective formation of granulomas was associated with a decrease in TNF production without an increase in IL-10 production.

### Gene Expression Programs and Deficient Granuloma Formation in Infected Patients

**Figure 5:**
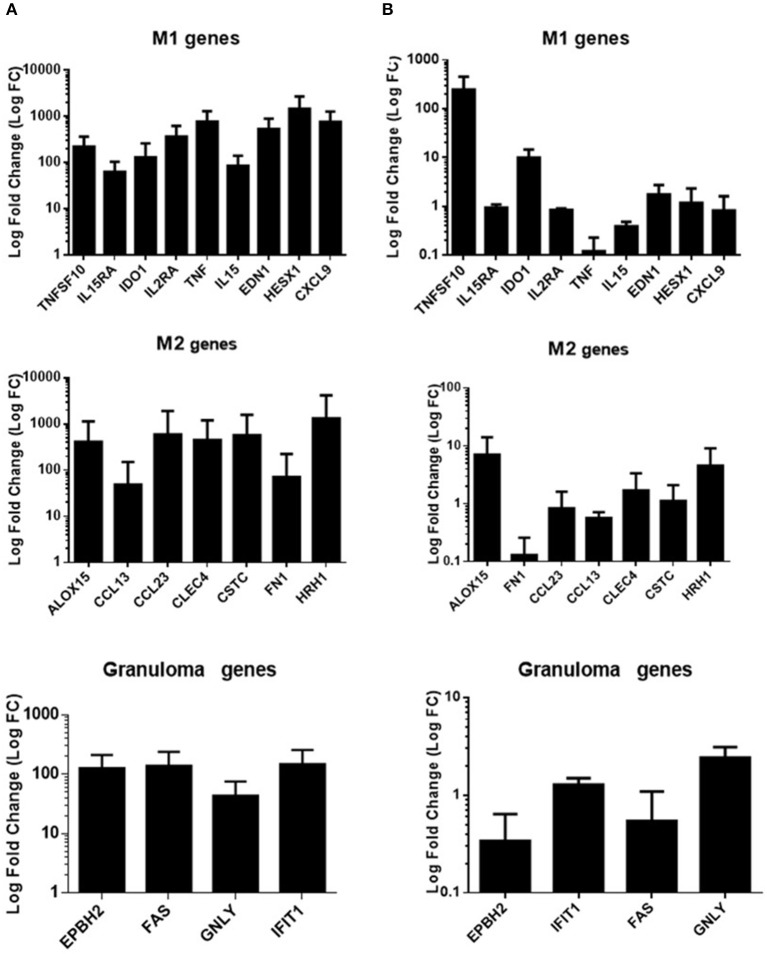
Gene expression program in infected patients. qRT-PCR was performed on a panel of M1, M2, and granulomatous genes. Gene expression was analyzed in cells from granulomas after dissociation in eight individuals (5 were infected) who formed granulomas **(A)** and in mononuclear cells in three patients who did not form granulomas (2 were infected) **(B)**. PBMCS were cultured without coated beads (unstimulated cells) and were used as control for fold change calculation. The results were normalized with the housekeeping gene β-actin. The FC of the target genes relative to β-actin was computed using the formula FC = 2^ΔΔCt^, where ΔΔCt = (Ct_Target_ - Ct_Actin_)_stimulated_ - (Ct_Target_ - Ct_Actin_)_unstimulated._ The results are expressed as the log fold change ± SD.

As the alteration in granuloma formation was related to TNF deficiency, we investigated the activation status of granulomatous cells in individuals who formed granulomas. We collected cells from granulomas after dissociation in eight patients who formed granulomas (5 infected patients and 3 controls) and assessed the expression of a panel of nine M1, seven M2, and four granulomatous genes, as reported previously (Naranbhai, 2016). In patients who formed granulomas, M1, M2, and granulomatous genes were upregulated, suggesting strong activation of granulomatous cells without M1 or M2 polarization (Figure 5A). It is noteworthy that in patients who did not form granulomas (*n* = 3), the expression of M1, M2, and granulomatous genes was markedly depressed, with the exception of TNFSF10 (Figure 5B). Hence, the granulomas formed in healthy individuals and infected patients exhibited normal activation profiles. Uninfected and infected patients with impaired formation of granulomas are associated with a profound alteration in the activation program of immune cells.

## Discussion

Elderly patients are susceptible to infection. Several factors affect the prognosis of infected elderly patients such as immunosenescence-associated immunosuppression, age-related organ changes, comorbidities, undernutrition or polypharmacy (Tannaou et al., 2019). A major risk factor seems also to be a pre-existing immune dysfunction. We hypothesized that the efficiency of mononuclear cells from infected patients to form granulomas *in vitro* was lower compared to elderly controls and we proposed answering this question by measuring the *in vitro* formation of granulomas using beads coated with BCG (Puissegur et al., 2004) and CB (Delaby et al., 2012) extracts. The choice of this model was only intended to explore the overall immune response to pathogens at a given time, which is difficult with other cell-mediated immune assays.

We showed that granuloma formation was impaired in infected patients compared to uninfected controls in response to both BCG and CB extracts. To exempt our data from the constraints of prior immunization, we compared granuloma formation in response to BCG to which a majority of individuals are immunized and CB to which a minority of individuals are immunized. Clearly, our results are not dependent on prior immunization as the modulation of BCG and CB granulomas was similar. This result is consistent with defective granuloma formation observed in patients with severe sepsis (Alingrin et al., 2016). Likewise, PBMCs from brain-injured patients with nosocomial pneumonia formed significantly fewer granulomas compared with brain-injured patients without nosocomial pneumonia and healthy donors (Deknuydt et al., 2013).

Deficient granuloma formation was observed in a large proportion (48%) of infected patients and a minority of uninfected controls (8%). No relationship was found between defective granuloma formation and age, sex or comorbidities. Clinically, only undernutrition was significantly associated with the impairment of granuloma formation. The causes of undernutrition in the elderly are varied and often multiple. They can be divided into three main types: medical, social, and psychological. Besides the causes of undernutrition unrelated to age (cancers, chronic inflammatory and psychiatric diseases for example), there are many risk situations that are more specific to the elderly. Aging is often associated with decreases in taste acuity and smell, deteriorating dental health, polypharmacy, cognitive disorders and less physical activity, which may all affect nutrient intake. Malnutrition can lead to a decrease in T cells. The number of CD4^+^ T cells from spleens of fasted mice is 40 to 50% lower compared to fed control animals (Saucillo et al., 2014). Other studies have shown that mice fed a protein-deficient diet exhibit lower T cell numbers compared to chow-fed control mice (Pena-Cruz et al., 1989; Taylor et al., 2013). A similar observation was made in humans. Malnourished children have lower circulating CD4^+^ and CD8^+^ T cell numbers compared to well-nourished children (Najera et al., 2004). The decrease in immune cell numbers during malnutrition contributes to a reduced ability of the immune system to mount a successful immune response to infection. This accounts for increased susceptibility to microbial pathogens such as influenza, *Mycobacterium tuberculosis, Streptococcus pneumonia*, and gastrointestinal infection microbes in malnourished individuals (Cegielski and McMurray, 2004; Verhagen et al., 2013). A relationship between defective granuloma formation and monocyte and lymphocyte counts has been reported in the absence of undernutrition. In patients with severe sepsis, the defective granuloma formation was associated with monocytopenia, whereas brain-injured patients experienced lymphopenia with a non-significant trend toward a lower lymphocyte count in patients with nosocomial pneumonia compared with patients without infection (Deknuydt et al., 2013; Alingrin et al., 2016).

We showed previously that monocytes migrate to the beads, mature into macrophages, then progress to epithelioid cells and MGCs under the influence of lymphocytes (Delaby et al., 2010). We found that when mononuclear cells did not form granulomas, neither epithelioid cells nor MGCs were found. When mononuclear cells formed granulomas, the number of epithelioid cells and MGCs in infected elderly patients was lower than in granulomas of controls. This alteration of granuloma organization is consistent with other reports. In brain-injured patients, the percentage of MGCs was lower in patients, especially with infection, compared with healthy donors (Deknuydt et al., 2013). These results suggest that the maturation process of macrophages is impaired in infected patients.

As many reports have established a relationship between cytokine production and granuloma formation, including the presence of epithelioid cells and MGCs, we investigated pro/anti-inflammatory cytokine imbalance. TNF is necessary for the formation of granulomas and Il-10 is involved in the inhibition of macrophage activation and high levels are associated with a disorganization of granulomas (Boomer et al., 2014; Pagán and Ramakrishnan, 2018). We found that TNF release decreased in PBMCs from patients unable to form granulomas with no increase in IL-10, suggesting that the modulation of TNF production was not related to the overproduction of IL-10. Similar results were obtained in patients with severe sepsis (Alingrin et al., 2016). Several studies have highlighted through the use of TNF deficient mice or anti-TNF-α drugs that TNF-α is essential for the formation and maintenance of granulomas (Kindler et al., 1989; Roach et al., 2000). Recently, we showed that etanercept (a fusion protein of IgG1 Fc domain and the extracellular ligand-binding portion of the human p75 TNF receptor) slightly delayed the formation of granulomas and reduced the generation of MGCs by inhibiting cell fusion in the same way as adalimumab treatment (a human monoclonal anti-TNF-α IgG1) (Mezouar et al., 2019). As the transformation and fusion of macrophages require autocrine stimulation by TNF production, the low production of TNF can explain the defective differentiation of granuloma macrophages we observed (Takashima et al., 1993).

We previously reported that BCG- and CB-induced granulomas were characterized by the expression of genes related to M1 macrophage polarization and chemotaxis (Faugaret et al., 2014). First, we found that polarization and granuloma formation-associated gene expression was dramatically decreased in mononuclear cells that did not form granulomas. M1, M2, and granulomatous gene expression was upregulated without polarization in patients who formed granulomas. The lack of polarization may be related to immunosenescence.

Altogether, we have shown that infection in elderly patients decreases granuloma formation and reduces the production of TNF-α and the formation of epithelioid cells and MGCs (Supplementary Figure 1). This study also suggests that the impact of infection on the granuloma formation is heterogeneous in elderly patients. Further studies are necessary to understand why some patients form granulomas and others do not when they are infected.

## Data Availability Statement

The raw data supporting the conclusions of this article will be made available by the authors, without undue reservation, to any qualified researcher.

## Ethics Statement

This study was approved by regional ethic committee Sud Méditerranée I. All the methods described were in accordance with the Declaration of Helsinski and national and international standards. The patients provided their written informed consent to participate in this study.

## Author Contributions

AD and J-LM designed the study. AD, JA, CC, and AB carried out the experiments. AD, BC, PV, and J-LM carried out the statistical analysis and drafted the manuscript. All authors read and approved the final version of the manuscript.

## Conflict of Interest

The authors declare that the research was conducted in the absence of any commercial or financial relationships that could be construed as a potential conflict of interest.

## Supplementary Material

Supplementary Figure 1 Summarizes the main results of the study. Most healthy elderly patients (92%) were able to form granulomas compared to only 48% of infected elderly patients. Granulomas typically comprise epithelioid cells and multinucleated giant cells. The impairment of granuloma formation was associated with reduced production of TNF without overproduction of IL-10. All genes specifically modulated in granulomatous cells were down-modulated in patients with defective granuloma formation.
