# Supplementary figures and images for: Defective Granuloma Formation in Elderly Infected Patients
Source: Front Cell Infect Microbiol. 2020 Apr 29;10:189. doi: 10.3389/fcimb.2020.00189 (PMC7201002; doi:10.3389/fcimb.2020.00189)

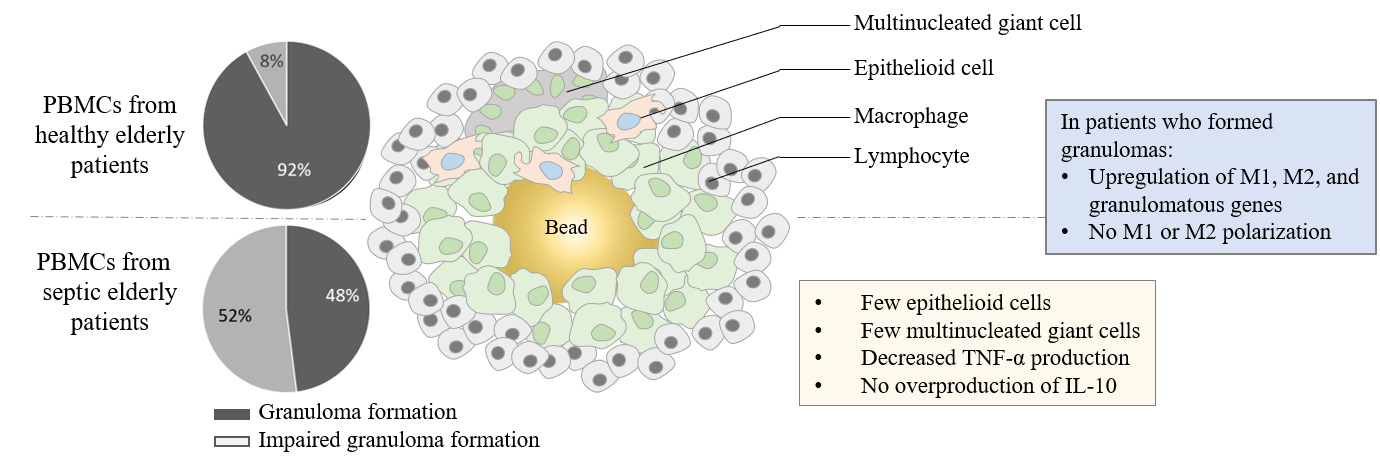

Supplement: Supplementary Figure 1 — Summarizes the main results of the study. Most healthy elderly patients (92%) were able to form granulomas compared to only 48% of infected elderly patients. Granulomas typically comprise epithelioid cells and multinucleated giant cells. The impairment of granuloma formation was associated with reduced production of TNF without overproduction of IL-10. All genes specifically modulated in granulomatous cells were down-modulated in patients with defective granuloma formation. [file Image_1.png]
